# Beliefs and practices of diabetic patients in Vhembe district of Limpopo Province

**DOI:** 10.4102/phcfm.v8i2.949

**Published:** 2016-05-20

**Authors:** Hilda Shilubane, Lizzy Netshikweta, Tshinyadzo Ralineba

**Affiliations:** 1 Department of Advanced Nursing, University of Venda, South Africa

## Abstract

**Background:**

Diabetes mellitus (DM) is a chronic condition affecting over 18 million people worldwide. It can lead to debilitating complications and premature death if not effectively controlled. South Africa, like any sub-Saharan countries and the world at large, is no exception. The prevalence of diabetes among South African adults has increased by 50% from 2009 to date, and an increase of some 11 million new diabetes diagnoses is expected by the year 2020.

**Purpose:**

The purpose of this study was to describe the beliefs and management practices of patients with DM in Vhembe district, Limpopo province.

**Setting:**

The study was conducted at Vhembe district clinics.

**Methods:**

A probability, purposive sampling was used to sample 100 diabetic patients. Data were collected using a pre-tested questionnaire. Data were analysed using the Statistical Package for Social Sciences version 19.0. Descriptive statistics, frequencies, and percentages were used to summarise the data from the study.

**Results:**

The majority of the respondents had poor management practice of feet care and annual eye examinations. Twenty four (24.0%) of the respondents believed that DM can be cured and 22 (22.0%) did not believe that diet helps in the management of DM.

**Conclusion:**

The belief that DM is curable can have a negative effect as patients can quit taking treatment once the disease is under control. This happens irrespective of the national guidelines for the management of DM. Therefore, some strategies should be sought that could enhance the implementation of the guidelines in order to combat the disease.

## Introduction

During the last 10 years, the prevalence of diabetes mellitus (DM) has increased dramatically in many parts of the world. The disease is now a worldwide public health problem, particularly in the developing countries, including the Republic of South Africa (RSA). The total number of people with diabetes worldwide is projected to rise from 171 million in 2010 to 366 million in 2030. In South Africa (SA), at the beginning of 2011 approximately 1.9 million people had been diagnosed with type 2 diabetes. The mortality rate is higher among people with diabetes than among the rest of the population, with an excess mortality of 45% in 2010.^1^ Excess mortality is mainly because of diabetes-related complications resulting from poorly controlled diabetes. The prevalence of DM in sub-Saharan Africa (SSA) is rising quickly.^2,3^ The condition was previously thought to be rare or not documented in rural Africa, but over the past few decades it has emerged as an important non-communicable disease.^4,5^

Mbanya et al.^2^ further indicated that the rate of undiagnosed diabetes is escalating in SSA countries, including SA, and people are at high risk of developing chronic complications, especially those not aware that they have the disease. SA, like any Sub-Saharan country and the world at large, is no exception. The prevalence of diabetes among South African adults age 30 years and above has increased by 50% from 2009 to date, and an increase of some 11 million new diabetes diagnoses is expected by the year 2020. DM is said to be the sixth leading cause of death and affects more than 5% of South Africans, and maintaining patients living with diabetes is costly.^6^

DM in SA is common and has emerged as an epidemic of the 21st century with the prevalence of adult onset diabetes ranging from 5.0% – 13.5% in the white population.^7^ Its prevalence among the black population is 6.0% – 8.0%, and 8.0% in the coloured population of Western Cape. Data from SA also show estimates of type 2 diabetes varying between 3.0% – 28.7% cases. Bradley and Puoane^8^ reported similar findings that the highest prevalence of type 2 DM was found among the Indian communities with 13.0% and the coloured communities of Cape Town with 28.7%. Similar to other countries, type 2 DM is more common among the 30 year age group and above.^9^ According to Mash^10^, the South African population diagnosed with diabetes were predominantly rich people living in urban areas; however, diabetes is becoming prevalent in rural areas because of westernisation, and threatens to overwhelm the health care system in the near future.

Diabetes is responsible for about 6000 deaths per year in SA. Many of the deaths occur among middle-aged people and could be prevented.^11^ In their study, Bradley and Puoane indicated that diabetes constitutes a significant health problem in SA. To curb this scourge, lifestyle modification and aggressive treatment for high risk individuals are required to prevent diabetes as well as delay the onset of its complications. In addition, an Indian study by Kheira et al.^12^ revealed poor adherence to treatment regimens as a result of poor attitudes towards the disease coupled with poor health literacy among the general public.

Hughes et al.^13^ conducted a study to assess community health workers (CHW) knowledge and beliefs in managing diabetes and they found that there was an agreement among CHW that people with DM believed to have been witched when they experience itching of the body. They further thought that a snake was eating the vagina when itching extended to the vagina.

According to Kiawi et al.^14^ most participants believed that the right place to seek treatment for their health, including diabetes care, was a modern health care facility.^5^ On the contrary, some people seek alternative treatment from traditional healers and other sources because they cannot afford to pay the hospital bills. This is affirmed by Peltzer et al.^15^ who found people to have different practices in managing DM. A considerable number of patients (80.0%) were found to treat their diabetes with western medicine and 20% used traditional medicine of which 2% use ‘Moshunkwane’ (chewed leaves of a tree) especially when they feel dizzy. They also believe in using ‘African Potato’ (root boiled and drunk while still hot and ’Lavita’ (herbal tea boiled and drunk when cool). Patients mentioned that these home remedies were better than western medication because they do not cause side effects like dry mouth. Furthermore, traditional medicine played an important role in diabetes care, and Lavita was seen as all-purpose remedy.^16^

In the study conducted in a rural hospital of SA, 20.0% used traditional medicine for diabetes management. Peltzer et al.^17^ in their study about the concept and treatment for diabetes among traditional and faith healers in the Limpopo province, SA, found that 92.0% of the traditional healers and 90.0% of the faith healers indicated that DM is curable and they used prayer, diet, and herbs.

Another study found that some people do not use tablets because they believe it causes kidney disease as it builds up in the body.^18^ Those who do not take treatment take other mixtures such as aloe, camphor, and boiled guava leaves, mixed with other substances. People believed in this mixture because they grew up drinking it as part of their culture. Furthermore, Maduna discovered that some western treatment was combined with traditional treatment, other people stop using treatment for some time while trying the mixture that a neighbour or a friend has advised them to use, but after some time the body gives a signal to go back to treatment.

SA has national guidelines for the management of DM, and the implementation of these guidelines appears to be poor. This has been seen by the low proportion of patients who have had examinations specific to identifying the occurrence of diabetes-related complications and those who have been referred to specialists like dieticians and ophthalmologists.^19^ In Vhembe District, which is one of the districts in Limpopo Province, patients with DM receive free medications in all health facilities. Nevertheless, adherence to medication remained ineffective in spite of free and available diabetes medication. DM morbidity and mortality rates may not decrease unless beliefs, attitudes, and proper management practices of patients with diabetes are maintained. ‘Cultural beliefs can be barriers to effective diabetic management as they greatly influence the perception of body image, value system, symptoms identification and interpretation in response to body dysfunction, hence care seeking behaviour that they would embark on’.^20^

## Purpose

The purpose of the study was to explore and describe the beliefs and management practices of patients with DM in Vhembe district, Limpopo province.

## Objectives

To describe the beliefs and management practices of patients with DM.

## Significance of the study

The results of this study could add to the existing body of knowledge regarding DM in Vhembe district as well as the entire RSA. Based on the information gathered on this research project, recommendations could be made for the development of health promotion activities and health education material needed by diabetic patients and the community at large. The researcher could consider the results to have a basic understanding of patient’s beliefs and practices regarding DM. Finally, these research findings could yield to further research.

## Research methods and design

### Research design

The study was conducted in Vhembe District clinics, Limpopo province, SA, with focus on Thulamela municipality region. A quantitative cross-sectional descriptive design was utilised to collect data using a pre-tested structured questionnaire among diabetic patients from three randomly sampled health care clinics.

### Population and sample

A probability systematic random sampling technique was used to select the respondents and simple random sampling was adopted to select the clinics. The population for this study included all patients suffering from DM who were on medication at the sampled health care clinics in Vhembe district of Limpopo province, SA. The study sample was 100 diabetic patients.

### Data collection

Ethical clearance to conduct the study was obtained from the University of Venda Ethics Committee and permission to conduct the study was granted by the Provincial Department of Health and operational managers of the institutions where the study was conducted. Informed consent was sought from diabetic patients and a self-administered questionnaire was used to collect data from respondents aged 40 years and above. The researchers personally delivered the questionnaire to diabetic patients in selected clinics.

### Data analysis

The data were coded, categorised, and entered into the SPSS software version 19.0. Descriptive statistics were applied and the frequency, percentages, and means of response were reflected. Cross-tabulation analyses and the Pearson’s chi-squared tests were done to evaluate the association and the strength relationship between variables. Findings are presented using tables and pie charts to enhance interpretation.

### Measures to ensure validity and reliability

Validity was ensured by giving the questionnaire to supervisors, statisticians, and expects in diabetes clinics who critically reviewed and verified the interpretations of the questions before being finalised for data collection. In addition, questions were formulated in simple language for clarity and ease of understanding. The questionnaire was then piloted to 10 diabetic patients who possessed the same characteristics as the study group and were excluded in the main study to ensure reliability. During piloting the researcher did not influence the completion of the questionnaire and the respondents were all exposed to the same questions.

## Results

### Demographic information

One hundred respondents completed questionnaires and their background information is summarised in Table 1. The respondents were grouped into four age categories: 38 (38.0%) of the respondents were in the age group of 40–49 years; 32 (32.0%) of the respondents were in the age group of 50–59 years; 24 (24.0%) of the respondents were in the age group of 60–69 years; and 6 (6.0%) of the respondents were in the age group of 70 years and above. Thirty-nine (39.0%) of the respondents were male and 61 (61.0%) were female. The majority 56 (56.0%) of the respondents were married and 18 (18.0%) were single. A minority 9 (9.0%) of the respondents were divorced and 17 (17.0%) of the respondents were widowed.

**TABLE 1:** Socio-demographic information of the respondents (*N* = 100).

Background characteristics	Frequency	%
**Age (years)**		
40–49	38	38.0
50–59	32	32.0
60–69	24	24.0
70 and above	6	6.0
**Gender**		
Male	39	39.0
Female	61	61.0
**Marital status**		
Married	56	56.0
Single	18	18.0
Divorced	9	9.0
Widowed	17	17.0

*Source*: Ralineba 2014

### Respondents’ religious status

Table 2 displays distribution of the respondents according to religious status.

**TABLE 2:** Respondents’ religious status (*n* = 100).

What is your religion	Number	%
Christianity	54	54.0
Tradition	42	42.0
Muslim	2	2.0
Other	2	2.0
		
**Total**	**100**	**100.0**

*Source*: Ralineba 2014

### Belief regarding cure for diabetes mellitus

**FIGURE 1:**
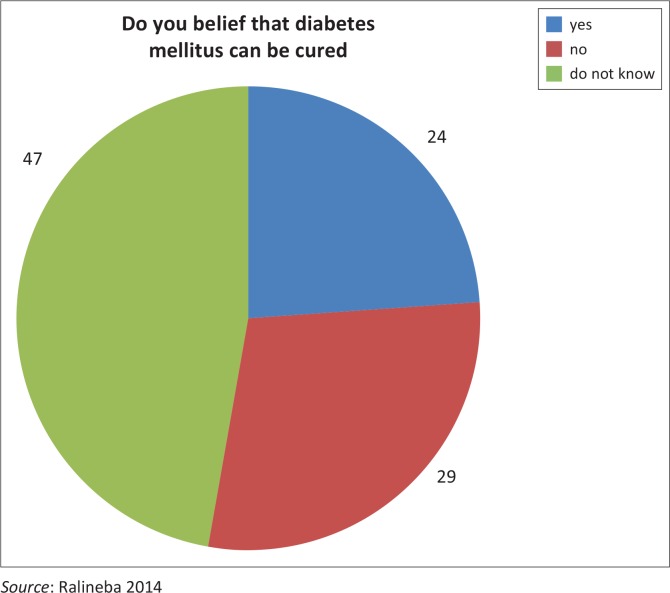
Response to the question if diabetes mellitus can be cured (*n* = 100).

Figure 1 indicates beliefs in percentages whether DM can be cured or not. Twenty-four (24.0%) of the respondents believed that DM can be cured, whereas 29 (29.0%) of the respondents reported that they did not believe that DM can be cured. As many as 47 (47.0%) of the respondents reported that they did not know whether DM can be cured or not. Therefore the participants’ beliefs could affect management of the disease.

### Belief that diet can manage diabetes mellitus

Table 3 displays beliefs that the respondents have if diabetes can be managed by diet. Out of 100 respondents, 42 (42.0%) believed that diet can manage DM. As many as 22 (22.0%) of the respondents did not believe that diet control can manage DM, whereas 36 (36.0%) did not know whether diet control can manage DM or not.

**TABLE 3:** Respondents’ Beliefs if diet control can manage diabetes mellitus (*n* = 100).

Do you believe that diet control can manage diabetes mellitus	Number	%
Yes	42	42.0
No	22	22.0
Do not know	36	36.0
		
**Total**	**100**	**100.0**

*Source*: Ralineba 2014

### Beliefs regarding benefit from physical exercise

The table above presents responses regarding beliefs on the benefit of physical exercise. Almost 41 (41.0%) of the respondents believed that they can benefit from physical exercise, whereas 15 (15.0%) of the respondents did not believe in physical exercise. As many as 44 (44.0%) did not have any information if diabetes patients could benefit from physical exercise (Table 4).

**TABLE 4:** Respondent’s beliefs if diabetic patient can benefit from physical exercise.

Do you believe that diabetic patient can benefit from physical exercise	Number	%
Yes	41	41.0
No	15	15.0
Do not know	44	44.0
		
**Total**	**100**	**100.0**

*Source*: Ralineba 2014

## Discussion

The study findings revealed that the respondents were type 2 diabetic patients suffering from DM for a period of 6 months and above. Out of the 100 respondents who participated in this study, the majority of the respondents were female. The majority of the respondents were in the age group of 40 to 69 years and were from rural areas as compared to few of the respondents who were from semi-urban and township areas. Almost half of the respondents did not have partners who actually served as support. According to some studies self-management behaviour is influenced by the type of support system the patient has.^9,21,22^ ‘Individuals are more likely to adhere to their diabetic health regimen if they have social support’.^23^ The number of respondents who believed in Christianity outweighs other religions and non-believers. It was important to enquire about religion because patients who are not affiliated with churches would opt to engage themselves in other practices as treatments for DM, such as the use of traditional medicines. Furthermore, studies show a positive correlation between less religious and good self-care ability. This was affirmed by Samuel-Hodge et al.^23^ and Chiou et al.^24^ who found most spiritual people suffering from type 2 DM to be having poor self-care compared to non-spiritual patients. Spiritual patients believed they could be cured without taking the prescribed medication as long as they pray to God. On the contrary non-spiritual patients believed in the health care provider’s advice and recommendations.

An encouraging part in this study was that the majority of the respondents were educated. This was affirmed by a study that demonstrated education of individuals with DM as very important.^25^ Educated patients are very receptive and understanding in terms of knowledge accumulation and ability to follow instructions. The majority of the respondents’ were either employed by the government or self-employed, a few were pensioners, and a minority were unemployed. Almost half of the respondents were suffering from both DM and hypertension.

Almost half of the patients were not sure whether DM can be cured or not while some believed that DM can be cured. This is supported by Coronado et al.^26^ who found that Mexican American diabetes patients believed that type 2 DM is a curable condition when treated by natural therapy rather than western medicine. In addition, Viral et al.^27^ also reported that 38.3% of the participants believed that DM was a curable disease especially when they used bitter substances. This belief can have negative impact on the management of the disease whereby patients can stop taking the medication when the disease is under control thinking that it is cured.

The majority of the respondents did not believe that diet control can assist in the management of DM, which could negatively affect adherence to diet regimen. On the contrary, control of DM can be better maintained if patients adhere to diet, prescribed treatment regimen, exercise, and compliance to appointments.^28^

More than half of the patients did not believe that physical activity could benefit them. On the contrary, physical exercise is important because of its effect of lowering blood sugar levels and reducing cardiovascular risk factors; whereas physical inactivity increases the risks of type 2 diabetes.^29^ This statement is also affirmed by Sellers^30^ who demonstrated the importance of exercise as ‘minimising abnormal stresses placed on the body and helping individuals to cope with and adapt to daily living and has psychological benefit such as promoting health and wellbeing’. In addition, ‘local behaviour practices and beliefs, as well as community specific external and internal barriers to change contribute to prevalence of type 2 DM, by promoting non-participation in physical activity’.^31^

Findings of this study revealed better practices of visiting health care clinics, which is supported by Shilubane et al. who found in their study of patients’ and family members’ knowledge and views regarding DM and its treatment that the majority of diabetic patients and their family members did not believe in traditional healers for the management of DM. On the contrary, participants believed in home remedies and reported problems to the clinic when at an advanced stage.^32^

## Limitations

Because this study was conducted at three health care clinics, the results did not reflect the beliefs and management practices of the general population of diabetic patients. More studies are required, particularly in different settings, to generalise the findings to the entire diabetic population.

## Recommendations

It is recommended that the directorate for chronic diseases and geriatrics (CDG) should encourage the supply of pamphlets to health facilities as a method of information dissemination using local language. The government should come up with strategies to enhance the implementation of the national guidelines for the management of DM. In addition, media (radio and television) should be used to convey diabetes messages to the entire society. In-service training and workshops should be organised for health care providers to keep them updated on new developments regarding DM in order to feel confident giving health education to the public. Patients need to be informed timeously about the danger and/or complications of uncontrolled diabetes; and nurses must refer or make appointments for diabetic patients to be seen by an ophthalmologist annually for eye screening and glaucoma evaluation.

## Conclusion

SA has national guidelines for the management of DM but the majority of diabetic patients still experience diabetes-related complications. Furthermore, patients with DM still hold beliefs about the disease which may be detrimental to their health as patients can quit taking treatment once the disease is under control, believing that the disease is cured.
